# Peer teacher training for health professional students: a systematic review of formal programs

**DOI:** 10.1186/s12909-018-1356-2

**Published:** 2018-11-15

**Authors:** Annette Burgess, Deborah McGregor

**Affiliations:** 1 The University of Sydney School of Medicine, Education Office, Faculty of Medicine and Health, Sydney, Australia; 2 Sydney Health Education Research Network (SHERN), The University of Sydney, Sydney, Australia

## Abstract

**Background:**

Skills in peer teaching, assessment and feedback are documented internationally as required graduate attributes for health professional students, placing emphasis on universities to prepare health professional graduates with teaching skills. The aim of this systematic review was to determine the rational, design, content and evaluation of student peer teacher training skills programs across the health professions.

**Methods:**

In October 2017, a search was conducted of five databases (Pubmed, Embase, CINAHL, ERIC and Cochrane Collection) using combinations of key search terms: ‘Student as teacher’, ‘near-peer teaching’, ‘student teacher’, ‘peer teacher’, ‘peer-to-peer’, ‘undergraduate’, ‘medical education’, ‘curriculum’, ‘program’, ‘training’, ‘allied health’, ‘health science’, ‘pharmacy’, ‘nurse’, and ‘medicine’, with results restricted to articles published in English within the decade. Articles were excluded if they were not original research, focused on a teaching approach other than peer assisted learning or teaching, did not adequately describe a student teacher training component of at least 3 hrs duration, or addressed only clinical skills training and not teaching skills training.

**Results:**

The two authors independently assessed 42 full-text articles for eligibility, with 19 articles satisfying criteria for inclusion. Dominating results were uni-disciplinary, faculty-led, non-mandated programs, targeting participants in senior years of training. Medicine was the dominant profession, with an obvious underrepresentation of the other health professions. Common program content included the foundations of education theory, teaching methods and techniques, and providing feedback. Summary and comparison of program design is restricted by gaps and inconsistencies in reporting, while the evaluation of programs remains largely subjective.

**Conclusions:**

Teaching is increasingly recognised as a core professional skill across the health workforce, with expectations to teach peers and colleagues, within and across professional disciplines, as well as to educate patients. Students, faculty and institutes may benefit from training programs being designed for implementation in any health profession; and further to this, implemented within an interprofessionally context. Consistent reporting of teacher training programs, and objective methods of evaluation would enable more in-depth investigation.

## Background

Skills in peer teaching, assessment and feedback are documented internationally as required graduate attributes for health professional students. For example, The Australian Medical Council requires a graduate to “*Demonstrate lifelong learning behaviours and fundamental skills in educating colleagues*” [1] (p4); the United Kingdom’s General Medical Council (GMC, 2009) requires a graduate to *“Function effectively as a mentor and teacher including contributing to the appraisal, assessment and review of colleagues, giving effective feedback, and taking advantage of opportunities to develop these skills*” [2] (p27); and the Pharmacy Board of Australia requires graduates to “*formally educate and train students and healthcare colleagues*” [3] (p109). On graduation, health professionals are expected to supervise, teach, facilitate, assess and provide feedback to colleagues, not only within their own discipline or profession, but also across disciplines within health [4]. Yet, despite being increasingly listed as graduate attributes, teacher training programs are rarely embedded as a requirement within university healthcare curricula.

Teaching skills are best acquired through a sequence of *training, practice* and *feedback* [5]. Reports of both informal and formal *peer assisted learning* (PAL) activities encouraging practice in peer teaching and assessment within the health professions are widely published, particularly within medicine [4]. However, there is a paucity of reporting on *training* provided to student teachers across the health professions*.* That is, it is unclear how health professional students are prepared for participation as ‘peer tutors’ or ‘peer assessors’ in peer assisted learning activities [4], involving teaching and assessment of clinical skills and procedural skills. Our recent systematic review of existing peer assisted learning activities within the single discipline of medicine found variation in the duration, timing, content, and mandate of training in preparation for PAL activities [4]. Further, where training was provided, assessment of students’ competence prior to engaging in peer teaching and assessment activities was minimal. In fact, the review revealed only one peer teacher training program that reported testing teacher competencies of the peer tutors prior to participation in peer assisted learning activities [4]. Importantly, this was also the only program reporting accuracy in peer tutor marking [6].

Common deficits in student teacher training programs have been highlighted previously, including: inadequate assessment of participants prior to participation as peer teachers; lack of practice opportunities; and lack of meaningful feedback to facilitate improvement in teaching skills [4, 5]. However, there is evidence to suggest formal training in teaching skills produces positive outcomes in terms of competency and further engagement in education. A number of studies have demonstrated improvement in participant teaching competence following participation in a teacher training program [5]. Darling-Hammond et al. assert, “It has been shown that students of certified (i.e., trained) teachers outperform those students taught by uncertified (i.e., untrained) teachers” [7] (p138). Studies have shown that graduates with prior educator training demonstrate greater teaching effectiveness and enthusiasm for teaching [8], including remaining active in teaching and pursuing more advanced teaching certificates [9], than those who did not partake in teacher training.

Despite the recognized importance of student teacher training, and increasing reports of single study interventions, there remains a lack of synthesized information on what a student peer teacher training skills program across the health professions should entail. To our knowledge, no previous systematic review has specifically investigated how faculties across the health professions (medical, dentistry, allied health, pharmacy, and nursing) provide teacher training for their students, and demonstrate important learning outcomes in peer teaching and assessment.

### Review objectives

This review aimed to answer the following questions:

- What is the rationale for implementing teacher training programs?
- What is the design and content included in the peer teacher training programs?
- How is student competence in peer teaching skills assessed?
- How are the teacher training programs evaluated?

## Methods

In October 2017, a wide literature search was conducted of five databases: Pubmed, Embase, CINAHL, ERIC and Cochrane Collection, using predetermined criteria. The search strategy applied combinations of key search terms, using 15 key words including: ‘Student as teacher’, ‘near-peer teaching’, ‘student teacher’, ‘peer teacher’, ‘peer-to-peer’, ‘undergraduate’, ‘medical education’, ‘curriculum’, ‘program’, ‘training’, ‘allied health’, ‘health science’, ‘pharmacy’, ‘nurse’, and ‘medicine’. Truncation was used where applicable to retrieve maximum relevant results, for example (students-as-teachers* OR peer teach*) AND teacher train* AND medicine. A total of 770 records were retrieved from the databases searches and citations were followed up from reference lists of retrieved articles, which yielded a further 16 results.

### Inclusion criteria

Only articles from peer reviewed journals were included. Included articles reporting on nursing, medicine, allied health, and pharmacy undergraduate or graduate entry health professional education were included. Only papers referring to the use of peer teacher-training programs in health professional programs of at least 3 hrs duration were included. Articles were only included where formal peer teacher training in preparation for clinical skills and procedural skills teaching were reported. The search was restricted to results published in English within the past decade. We chose to only include the past decade, since peer assisted learning has become increasingly formalised in recent years [4], and as such, it is likely that training programs have also become more formalised.

**Fig. 1:**
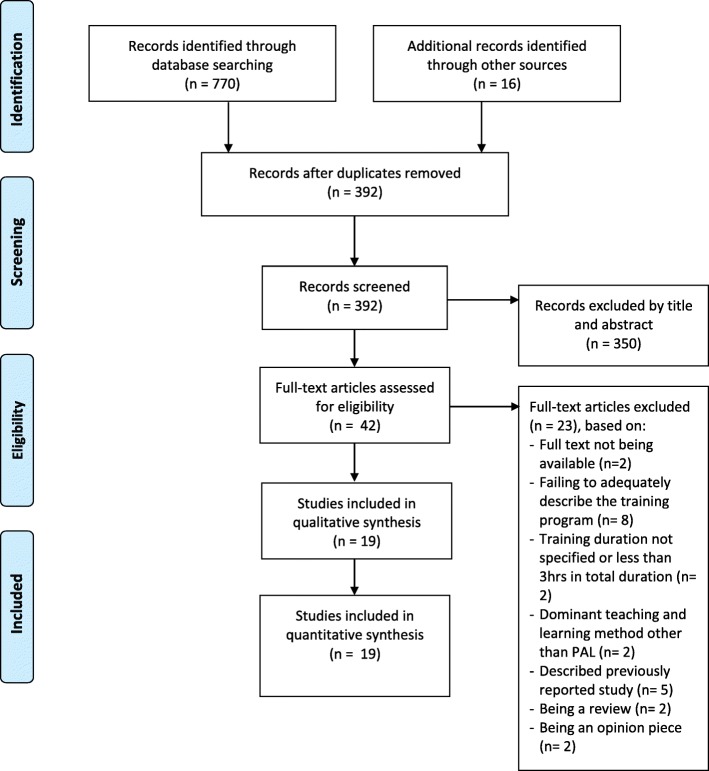
PRISMA description of literature search results

A bibliographical management program (Endnote X7, Thomson Reuters, New York) was used to construct a search library. The characteristics of the literature search are summarized in Fig. 1.

### Exclusion criteria

Articles were excluded if they were not original research, focused on a teaching approach other than peer assisted learning or teaching, did not adequately described a student teacher training component of at least 3 h duration or addressed only clinical skills training and not teaching skills training. Articles were excluded if they failed to adequately describe the training program provided to peer teachers. Articles were excluded if their focus was on subject or clinical skill specific content to be taught (e.g. anatomy and dissection skills) without specifically preparing participants with pedagogical knowledge and skills for teaching delivery. Papers were also excluded if they focused on the provision of feedback after a student teaching episode, without formal training before. Studies where the same peer teacher training activity had been previously reported were removed.

Reviewer DM conducted the initial title and abstract review of all results, with AB providing a second review of any results in question of inclusion. Independent full text review was conducted by both authors, with any disagreements resolved through face-to-face joint review and discussion. A data extraction tool was constructed by authors and applied to the analysis of results.

### Ethics approval

No ethics approval was required for this systematic review.

## Results

The abstract review yielded 42 articles for independent full text review by authors DM and AB, with 19 articles meeting the criteria for inclusion in the final analysis.

### Context

The literature contained global examples of student teacher training programs. Programs were implemented in the United States of America (USA) (7/19) [10–16], Germany (4/19) [17–20], Australia (4/19) [21–24], Netherlands (1/19) [25], United Kingdom (UK) (1/19) [26], Canada (1/19) [27] and Israel (1/19) [28]. One program was student led [26], implemented by the Junior Association for the Study of Medical Education (JASME), a UK student run organization. Three results involved a student/faculty collaboration for program implementation (3/19), including the program described by Smith et al., which involved a collaboration between the American Medical Student Association and the Mount Sinai School of Medicine [15]. All other program implementations were led by a university faculty member(s).

Results largely reported on teacher training interventions involving student participants from a single academic site. Three studies drew participants from multiple academic institutions or clinical practice sites, including student participants invited from: multiple clinical school sites at the University of Sydney [24], all allopathic and osteopathic medical schools in the USA and Canada [15], and from institutions across the USA to attend a national conference [10].

### Rationale

A common theme related to the understanding that students will be required to teach in their future careers and, therefore, the reason for implementation was the provision of early opportunities for students to prepare for teaching roles [11, 16, 27]. A lack of current teacher training initiatives for students was cited as a reason for implementation [12], as was the belief that training in the fundamentals of teaching would be well received by students [26] and optimise learning [12, 22]. Blanco et al. hypothesised that students may become better learners and enhance their communication skills as a result of teaching training and experience [11]. Programs were also implemented to help overcome problems associated with a shortage of teaching resources [28] and curriculum uniformity [18]. The program description provided by Dickman et al. acknowledged a need to overcome a shortage of qualified anatomy dissection instructors within the newly formed Faculty of Medicine as a driving force for the initiation of a near-peer teaching program to train such instructors [28].

### Program description

Globally, a variety of programs have been designed, adopted and adapted, aimed at providing students with opportunity to develop and improve teaching skills. Smith et al. outline the implementation of the ‘Training Tomorrow’s Teachers Today’ (T4) program, an annual week long national teaching and leadership retreat [15]. Students (*n* = 23) spanning years two through four of medical school participated in daily 6-8 h interactive classes, plus evening readings, video reviews and teaching session preparation. An adaption of the T4 program was implemented by Andreatta, who again brought together students (*n* = 13) with demonstrated interest in teaching and leadership experience from across the USA for a five-day intensive conference [10]. One study implemented the ‘Teaching on the Run’ (TOR) program [29], a six module program originally conceived to train clinicians to teach students and junior doctors, while two papers report on modified TOR programs [23, 24]. A summary of student teacher training programs is provided in Table 1, including outline of the Teaching and Learning Communication Skills (TALKS) program, a longitudinal multimodal program for year 4 medical students [12], and the Student-Teacher Education Programme (STEP), an extra curricula experience for students in their final year of medicine [13].

**Table 1:** Summary of student teacher training programs

Study	Country / Context	Program description	Curricula	Group size	Participant program, level	Led by	Frequency, duration & mode
1. Andreatta et al., 2009	USA, University of Michigan Medical School	Adaption of the ‘Training Tomorrow’s Teachers Today’ (T4) conference	Extra curricula	13	Medicine, year unspecified	Faculty	5 days, F2F Conference
2. Blanco et al., 2014	USA, Tutfs University	Student-as-Teachers (SAT) program	Core curriculum	–	Medicine, years 1–4	Faculty	4 yrs., Four online modules (40-60 min each), 25 h practical field experience
3. Blatt & Greenberg, 2007	USA, George Washington University	Teaching and Learning Communication Skills (TALKS) program.	Elective	28	Medicine, year 4	Faculty	Six 2.5 h workshops, a practicum, and service as standardised patient.
4. Burgess et al. 2012	Australia, University of Sydney	Implementation of Teaching on the Run program (TOR)	Extra curricula	17	Medicine, year 3	Faculty	Six 3 h workshop sessions
5. Carr et al., 2016	Australia, University of Western Australia	Peer assisted learning (PAL)	Extra curricula	Varies	Medicine, year 6; Nursing, year 2; Podiatric, year 4; Pharmacy, year 2; Health Sciences, year 3; Dentistry, year 4.	Faculty	F2F training sessions between 1 and 4 h and practical opportunities
6. Erlich & Shaughnessy, 2014.	USA, Tufts University	Student–Teacher Education Programme (STEP)	Extra curricula	13	Medicine, year 4	Faculty	Training sessions prior (30 min) and post (45 min) peer teaching session, once a week × 12 wks
7. Dickman et al., 2017	Israel, Bar-llan University	Near-peer teaching program	Extra curricula	12–15/year	Medicine, year 1	Faculty	Eight sessions, large and small group training and advanced practical skills training.
8. Fellmer-Drug et al., 2014	Germany, Heidelberg University	Didactic student tutor training program	Extra curricula	Varies	Medicine, year unspecified	Faculty	Approx. 14.5 days across eight weeks, four F2F modules, including practical experience as peer tutor.
9. Gainor 2014	USA, Brown University	Medical Education Elective	Elective	12	Medicine, year 4	Faculty and student	One week (5 days), F2F didactic sessions and three wks practical
10. Newton & Wright, 2011	UK, The Junior Association for the Study of Medical Education (JASME)	‘ASME Teaching Toolkit for Medical Students’ one-day clinical teaching course	Extra curricula		Medicine, Senior year	Student	One day F2F workshop with plenary presentations & small group teachings.
11. Schuetz et al., 2017	Germany. Ludwig-Maximilians-Universität	Near-peer teaching program	Extra curricula	–	Medicine, any stage	Faculty	Two days initial intensive training with modules (20 lessons), intensive workplace-based training (20 lessons). Lesson = 45 min lecture or seminar
12. Silbert & Lake, 2012	Australia, University of Western Australia	Modified TOR for PAL program	Extra Curricula	29	Medicine, years 4–6	Faculty and student	Two 3 h small group interactive workshops
13. Smith et al., 2007	USA, The American Medical Student Association, with the Mount Sinai School of Medicine	Training Tomorrow’s Teachers Today (T4) national medical student retreat.	Extra curricula	23	Medicine, years 2–4	Faculty and student	One week, 6–8 h/day, interactive classes
14. Song, 2014	USA, University of Rochester	The Medical Education Pathway (MEP), longitudinal student-as-teacher program	Elective	15–20/year	Medicine, years 3–4	Faculty	Completed over 2 yrs., multimodal: 2 large lectures, 6 mandatory workshops, 1 elective workshops.
15. Van Diggle et al., 2015	Australia, University of Sydney	Student Teacher Training (TT) program	Extra curricula	23	Medicine, years 3–4	Faculty	two 3-h sessions over 2 days
16. Walser et al., 2017	Germany, Ulm University	Train the Tutor (TtT) to train student tutors as near-peer teachers	Extra curricula	23	Medicine, 5th semester of study and above.	Faculty	Workshop (16 units), practical phase (114 units – 25 days), advanced seminar (9 units)
17. Weyrich et al., 2009	Germany, University of Tu¨bingen	PAL tutor training	Extra curricula	14	Medicine, years 4–5	Faculty	Two 3-h sessions, plus a standardised student-tutor didactics seminar (two 4-h sessions)
18. Yeung et al., 2017	Canada, University of Toronto	Student as Teachers (SAT) program	Extra curricula	20	Medicine, year 2	Faculty	Seven months integrated into full academic year. Eight 2 h educational modules, five practical teaching sessions, and three independent assignments
19. Zijdenbos et al., 2011	Netherlands, Utrecht University Medical Center	START block: ‘Supervised Training in Attitude, Research and Teaching’.	Core curriculum	12–15	Medicine, year 6	Faculty	1wk course spread over 2wks (a total study load of 40 h)

### Participants - professional discipline

Medicine was the dominant professional discipline represented in the literature. All student teacher training programs reported included medical students in the cohort of participants. Two papers reported on programs involving multidisciplinary participation. Carr et al. list students from medicine, nursing, podiatric medicine, pharmacy, dentistry and health science as program participants [22]. While not clearly stated, the paper suggests that each of the disciplines participated in separate pilot implementations of the same program, rather than as a multidisciplinary cohort. In the study conducted by Walser et al. involving the preparation of student tutors as near-peer teachers (NPT) in a dissection course (DC), it is acknowledged that the DC course is taken by medical students and dentistry students, but description of the NPT cohorts suggests only medical student participation as student tutors [19].

### Participants - stage of training

The majority of programs (*n* = 13/19) targeted participation of student’s tutors in their senior years of study, typically years 3–4 of a four-year program, or from year 4 of a six-year program. One program implementation incorporated training at different stages throughout the four-year medical school training, commencing in year one [11]. Another program allowed students to commence as a NPT at any time of their study, after completion of the first preclinical semester [18]. Within this program, Schuetz et al. indicated that *“near-peer is not necessarily reflected by a higher semester of the student teacher compared to the tutee, but rather in a measurable knowledge advance of the student teacher, for example in terms of a successfully completed assessment or a successfully visited course in which the tutees have not yet successfully participated”* [18] (p73). Dickman et al., who introduced their program to first year students, did this largely out of necessity to rapidly prepare suitably trained student teachers to address teaching resource issues within a newly formed program [28]. Two programs involved medical students in their second year of training [15, 27]. The program implemented by Carr et al. involved second year students from nursing and pharmacy, along with students from multiple professional disciplines at more advanced training stages [22].

Teaching practice components of programs involved student learners, roles typically fulfilled by fellow program participants, student peers or near-peers from another stage of training. Analysis of the stage of student-teachers in relation to student-learners, sometimes referred to as the ‘distance’ between teachers and learners, was not a focus of this review.

### Recruitment

Recruitment of participants was largely associated with students’ expressed interest in teaching and leadership experiences [10, 14, 15, 21, 27, 28]. Some recruitment processes entailed program conveners disseminating email invitations to targeted cohort of students [21, 24]. Other programs recruited participants via nomination or specifically targeted invitation, such as invitations based on academic performance. For example, Dickman et al. invited students who graduated their anatomy class with a grade of 85% or above [28], while Smith et al. sought Dean nomination of one student from within each participating school [15]. Several programs involved a competitive recruitment process [10, 15, 16]. For example, the detailed two phase application process outlined by Song commenced in year two of the prospective participants’ studies and required students to firstly compose a statement of intent, identify a faculty mentor and obtain documented support of a dean. Following this, students compose a detailed application incorporating a proposed lecture and small-group lesson, a teaching requirement checklist, signed off by a faculty dean and mentor [16].

### Program content

Common topics addressed in formal training programs included, the foundations of education theory, including the principles of adult learning, teaching methods and techniques, and providing feedback. As outlined in Table 2 the common content topics include large group didactic instruction, small group facilitation, and how to conduct assessment and evaluation. One program included specific instruction on grading Objective Structured Clinical Examinations (OSCE) [14].

**Table 2:** Student teacher training program content

Teacher Training Content	Study reference
Education theory, including adult learning principles, educational psychology, motivation, educational research	10,11,13-15,25–28
Planning teaching, including developing curriculum, session planning, writing objectives and outcomes, topic specific preparation	14,16,17,19,23,25
Teaching methods and techniques, including clinical and practical skills teaching, providing explanation	10,15,19-21,23,24,26
Small group instruction and facilitation skills, including facilitating tutorials	10,13,14,16,17,24,25,27,28
Didactic and large group instruction, including presentation skills	10,14,16,17,19,22,28
The learning environment	11,15
Group and classroom management, including group dynamics, session control, coping with difficult classroom situations	10,15,19,20,23
Assessment and evaluation	10,11,14-17,21,24,28
Instructional design	10,11
Providing feedback	10,13-16,19,21–26,28
Communication	15,17,22
Reflection, reflective practice and self-evaluation	10,13,23,28
Leadership/mentorship/professionalism	10,13,14,17,26
Motivation and optimising learning	10,23,25
Writing assessments and exam questions	15,16,19

Programs often involved participants in planning for teaching episodes, including developing curriculum, writing learning objectives and outcomes, planning sessions and preparing for topic specific teaching. One program involved participants in curriculum design activities requiring the creation of video-recorded specialty-specific oral presentations for junior students to access electronically [14]. In another example one quarter of the program and homework time was dedicated to the students devising, piloting and evaluating a teaching project for implementation at their respective medical schools [10]. Two programs addressed the preparation of a teaching portfolio [16, 19].

Addressing teaching methods and techniques typically included opportunities to rehearse and practice clinical skills teaching and provide precise explanation. In one example, the program incorporated exercises with non-announced ad hoc teaching situations, providing participants with opportunity to rehearse realistic teaching situations [20]. Existing frameworks were used in many of the programs. For example, Peyton’s four step approach [30] to teaching a skill was utilised within several programs [17, 19–21], as was the Peyton’s set-dialogue-closure format [30] to plan and deliver teaching session [21, 25].

Other, less frequent, content topics included: involving patients in teaching [23], using simulation and standardised patients [10], program administration [10], promoting understanding and retention [15], basic acquaintance with medical education topics [25] and medical education journals [25].

### Program duration

The review excluded any teacher training program of less than 3 hrs total duration, therefore, all programs exceeded this minimum. The average duration of student teacher training programs, in terms of total number of hrs, is difficult to report on due to variability in reporting. Programs of the briefest duration included a multi pilot program combining 1–4 h of face-to-face training, with practical training of unspecified duration [22], and programs consisting of a limited number of intensive teacher training sessions, such as the two 3 h interactive workshops [23, 24]. Also contained within the results are examples of intensive one day [26] and week-long programs [10, 14, 15]. Zijdenbos et al. describe a one-week basic teacher training block, implemented over a two-week teaching period, representing a total of 40 h of study time [25].

Several longitudinal program implementations are described. Blanco et al. describes a program implementation across different stages of a four-year medicine program, during which students achieve twenty-eight learning objectives via a combination of online modules, each taking 40–60 min to complete (a total of 4 h), and a minimum of twenty five contact hrs of field teaching experience [11]. Song describes the ‘Medical Education Pathway’ (MEP), a structured extra curricula student-as-teachers program, which is completed over the senior two years of the medical program and involves a minimum of forty teaching contact hrs [16]. Two papers report on multimodal programs integrated into the full academic year, including the program described by Yeung et al., involving a total eight 2 h modules, five practical teaching sessions, and three independent assignments, implemented over seven months of the academic year [27], and Blatt & Greenberg’s program for year 4 of the medical students, involving six 2.5 h workshops, a teaching practicum and service as a standardised patient junior year examinations [12].

Programs involving participation in sessions over the course of a single semester, include a program requiring participant attendance at a seminar prior to assuming the NPT position, a monitored practical teaching phase, and concluding with attendance of an advanced seminar [19], and an eight week tutor training program, involving approximately 14.5 days of participation across four modules over the course of the summer semester [17].

One study [24] reported on the revision of the length of the program conducted by Burgess et al. [21], reducing the duration down from 3-h sessions over six evenings, to two 3-h sessions. Restricted participation in the lengthier program, due to significant time commitment, was cited as the reason for the revision.

### Extra curricula, elective or core curriculum

Two studies reported on the implementation of a program as core curriculum, a mandatory student-as-teacher (SAT) program for all students expanding across the four year medical curriculum [11], and the ‘Supervised Training in Attitude, Research and Teaching’ course (START-block), a one week basic teacher training course in the sixth and final internship year [25]. Three examples of elective programs were noted in the literature [12, 14, 16], while the remaining majority (14/20) reported programs involving extra curricula, voluntary participation.

### Size

It is difficult to report on the average number of participants, due to variability in reporting. Seven programs reported participant numbers ranging from 10 to 20 students [13, 14, 16, 20, 21, 25, 27], including programs reported by Song, with an average of 15–20 participants per year [16], and Zijdenbos et al., with an average of 12–15 participants per group, and conducted about 20 times a year [25]. Five programs reported between 21 and 30 participants [12, 15, 19, 23, 24]. The pilot programs reported by Carr et al. had variable numbers, ranging from *n* = 6 in a program for a podiatric medicine student cohort, up to *n* = 53 in a program for a medicine-paediatric student cohort [22]. The study reported by Fellmer-Drug et al. had 56 student tutor trainees enrolled in their extra curricula tutor training program at the time of reporting [17]. Schuetz et al. reported on a large scale implementation of a NPT program with the annual intake of in excess of 900 medical students, however, it is unclear how many students participated in the voluntary NPT training [18].

### Mode of delivery

Face-to-face delivery of content was the most common training mode, including both large group presentations, as well as small group training and practice sessions. Formats included of workshops or seminars [16, 19, 23, 26], out of class and evening training sessions [12, 21] and conferences [10, 15]. Several programs focused on didactic training sessions with integrated opportunities to practice teaching delivery and practical skills training with fellow participants [15, 21, 23, 24]. Other programs served the purpose of preparing students to assume a NPT role, and therefore didactic training preempted actual classroom or practice based teaching experiences [14, 19, 20].

Online learning materials were used as primary content delivery mode or to supplement face-to-face training. For example, Blanco et al.’s longitudinal program involved participants completing four self-directed online learning modules, one per year [11]. Audio-visual content, in particular pre-recorded video content, was frequently used for skill development [12]. In some cases, students were required to create video-recorded specialty teaching content [14] or as examples of teaching sessions [15]. Video recordings were frequently used as opportunities for student reflection and self-assessment [27], and the provision of feedback and coaching [10, 20]. For example, following independent student reflection of their recorded teaching session, Smith et al. used video review sessions as a platform for extensive peer and faculty feedback, from which students could create personal teaching goals [15].

### Incentive

The majority of programs specified no participant incentive. One study acknowledged that no incentive was provided, outside of inclusion of the experience in their CV when applying for post-graduate training positions [13]. A number of programs resulted in the award of a certificate of completion [17, 27] with one program counting as recognition of prior learning towards a Certificate in Academic Teaching at a related institution [17]. One study reported on contracted student assistants receiving financial compensation [20] and another noted that up to six of the best graduates of the elective training program, based on their peer evaluations, were offered a near-peer instructor position in the next academic year [28].

### Student assessment

Assessment methods typically included formative feedback on student’s teaching from faculty, experienced educators, peers and learners. Walser et al. note the use of 360-degree feedback system, inclusive of self-assessment, instructor and student perspective [19]. A similar combination of feedback methods applied by others [11, 13, 14]. In an example of peer-to-peer feedback, Dickman et al. made ongoing verbal and written peer evaluations a participant requirement, with each session evaluated by participant feedback rubrics developed by the students under staff supervision [28]. Assessment of student-teachers by student-learners was common where near-peer teaching occurred [11–14]. Other assessment items included formative quizzes [11], reflective field teaching experience reports [11, 18] and maintaining a teaching log [19]. One program culminated in formal project presentations by each student to their fellow attendees [15].

### Program evaluation

Studies commonly applied a mixed method research design, involving quantitative data captured through pre and post inventories and qualitative data captured through written comments, open-ended questions, semi-structured interviews &/or focus groups.

Reported evaluation items included: student satisfaction [28]; student opinion [12]; student’s perceived abilities [21]; perceived value of program [28]; perceived benefits [23]; perceived effectiveness [11]; student self-assessment of knowledge change [11]; self-evaluation of teaching [23]; student competence and performance [12]; program delivery [23]; and achievement of program outcomes [23].

One study included the quantitative and qualitative analysis of teaching logs written by the near peer teachers [19], while another included the tracking of student completion of modules and field teaching experience [11]. Burgess et al. used open-ended questions aimed at identifying motivating factors for participating in the peer teaching program and invited participants to a focus group aimed at exploring participant perspectives, attitudes, behaviours and experiences [21]. Carr et al. also utilised focus groups and interviews with students and staff, as well as direct observations of the peer teaching activity or fieldnotes [22].

Erlich et al. noted that they followed a modified Kirkpatrick’s hierarchy of curriculum evaluation [31]: Reaction, Learning, Behaviour, and Results [13]. This included analysis of student learners’ (first-year students) OSCE scores and mandatory electronic course evaluations. Gainor et al. also compared OSCE performances across two classes of junior students, as well as involving all 12 near-peer instructors in a three-hour debriefing session at the end of the course and obtaining anonymous, written feedback [14]. Weyrich et al., whose study involved a randomised trial to evaluate the effectiveness of peer-assisted learning in technical skills training, also analysed OSCE results to compare students assigned to peer-tutor-led, faculty-led and control, that is, no skills lab training, and randomised groups [20].

Three results referred to the use of longitudinal evaluation, including a follow-up survey at 11 months [15], a 2 year post intervention survey [10], and a post-graduate year 1 Residency Program Directors survey to assess former student’s teaching during internship [11].

## Discussion

This systematic review sought to investigate and report on the provision of teacher training programs for university students across the health professions, including medicine, dentistry, allied health, pharmacy, and nursing. We identified a total of 19 studies reporting on the implementation of teacher training programs. Below we discuss 1) the rationale for implementation of the programs; 2) the design and content of the programs; 3) assessment of student competence; and 4) evaluation.

### Rationale for implementation of teacher training programs

Key reasons for implementation of the student teacher training programs included the preparation of participants for immediate practice as student peer tutors, as well as preparing graduates with widely acknowledged requisite skills for future practice as healthcare professionals with teaching responsibilities. Although not reported in the reviewed papers, provision of program outcomes, clearly linked to professional association requirements, may reinforce the need for student teacher training programs. Several studies repeated a common assertion that the act of peer teaching and assessment can provide a rich learning experience, with the opportunity to revise and reflect on one’s own knowledge and skills [31], however, few provided objective measures to substantiate this claim. Although the majority of today’s healthcare curricula afford early clinical exposure for junior students, availability of hospital clinicians for teaching is usually limited.

The act of implementing training to overcome teaching resource capacity issues was the reasoning provided by the authors of a newly formed faculty [28], and perhaps justifiably so. However, part of the intention of peer teaching programs is to provide additional support to students, rather than alleviate teaching burdens on clinicians.

### Design and content of the programs

#### Discipline based

The majority of the teacher training programs occurred within medicine, with limited examples from other health disciplines. Although at times difficult to determine from the reporting, all programs were delivered as uni-professional training. This is somewhat surprising, given that evidence suggests that interprofessional education during healthcare training leads to improvements in leadership, collaboration and communication between healthcare teams, ultimately improving patient safety [32–34]. This link provides a powerful reason for implementing teacher training within an interprofessional context, rather than the individual discipline silos. While there are several recognised challenges to implementation of interprofessional curricula, such as healthcare professionals often preferring to teach within their own disciplines, and pragmatic issues, including the logistics of timetabling across disciplines, it is an area worthy of consideration in teacher training program design.

#### Participants

The review identified that the majority of programs target students in their senior years of studies. This aligns with claims that engagement in teaching activities in the senior years of university education, at a point in time when students have an understanding of their own professional responsibilities, may assist in development of professional identity [35]. Further to this, by their senior years, it is expected that students have a sound clinical knowledge and skills to draw on when teaching. Despite this, the literature contained several examples of programs implemented at various and more junior stages of studies, including examples of longitudinal programs spanning multiple years of a qualifying course, building student competence towards graduation.

While it has been suggested that peer teaching should be embedded within healthcare curricula as a means to increase its efficiency [36], the review found that the majority of programs were extra curricula activities, requiring voluntary participation. Additionally, several programs described a selective or competitive recruitment process [10, 15, 16, 28]. However, it has long been asserted that it is the weaker students who may benefit the most from participation as peer tutors [37]. Further to this, there is not always a correlation between facilitation skills and academic ability [38]. Given the aim of peer teaching is to implement mutually beneficial educational activities for all students [38], it may be unwarranted to exclude students on the basis of their academic performance; and justified to consider embedding student peer teacher training programs within curricula.

#### Method and time of delivery, and content

The duration of programs varied greatly from 3 hrs, to a longitudinal program spanning two years. Topics taught include teaching methods, educational theory, curriculum design, formative assessment and feedback. Many of the programs included aspects of online supplementary learning, out of class preparation and homework activities, methods common to ‘flipped classroom’ approaches, which have well documented advantages, such as the students attending class prepared to engage in activities [39]. The incorporation of the flipped classroom approach into program designs may assist in providing flexibility and maximising face-to-face training components, enabling greater participation of students with restricted availability due to demanding schedules. This was the experience for van Diggele et al., who reported limited teaching time due to restricted availability of students [24]. It has been suggested that the amount of training and design be dependent upon the requirements of individual tutoring activities and needs analysis outcomes [40]. For example, some suggest that training encompass not only development of teaching skills, but also content specific knowledge [38] dependent on the teaching or assessment topic. What is apparent from review of the literature, is the potential for the standardisation of programs with key design elements that enable greater consistency in teaching training experiences for students across the health professions, while maintaining the flexibility to customize programs to the specific audience requirements to strengthen student participation.

#### Opportunities for practice

Evidence suggests that active learning opportunities to engage participants, with multiple opportunities for practice with immediate feedback, provide a deeper understanding of knowledge, and assist knowledge retention [41, 42]. While all training programs included opportunities for participants to practice teaching skills, not all programs were integrated or linked with actual peer assisted learning sessions, where students assumed the role as peer tutors or assessors. Knowledge and skills acquired during initial training programs require ongoing reinforcement and practice, which may be best achieved by embedding and marrying more peer teacher training and PAL opportunities within curricula.

### Assessment of student competence

The terms ‘feedback’ and ‘assessment’ are often used interchangeably. However, distinct from assessment, feedback presents information, rather than judgement, forming an integral part of the learning process. Feedback from faculty or peers was common to teacher training programs, occurring both during practice opportunities within teacher training programs and PAL activities. The aim of feedback is for the recipient to reflect on their performance, and make improvements in order to reduce the gap between actual and desired performance [43]. The recipients’ perception of the quality of the feedback is therefore important in eliciting a positive attitude towards change, and both feedback by peers and faculty have been reported as valued by students [44]. It has been suggested that direct observation in the ‘work place setting’ offers the optimal setting for determining competence [45, 46], and this was reported in two studies, where teaching of near peers was directly observed by experienced educators [13, 14].

### Program evaluation

Program evaluation was largely linked to subjective measures, including student perception of change in teaching ability. Use of a standardized evaluation framework would promote and facilitate consistent reporting, allowing researchers to compare and contrast the effectiveness of programs**.** Additionally, greater use of objective measurement of student knowledge and skills in peer teaching and assessment would help to ascertain the effectiveness of programs. For example, Weirich et al., demonstrated, based on Objective Structured Clinical Examination (OSCE) results, that students taught by their peers trained in teaching injection technique, performed as well as those taught by academic staff.

#### Limitations

This systematic review has uncovered a number of gaps in reporting on peer teacher training programs, making it sometimes difficult to accurately interpret findings. There were limitations in the ability to report and compare program outcomes due to inconsistencies in study design and reporting.

## Conclusions

The increased availability of teacher training programs appears to have arisen due to the continued reference to teaching skills as graduate competencies; the educational benefits of engaging in teaching activities; and limited teaching resources within institutions. Encouragingly, opportunities for practice, with the use of feedback on performance formed an integral part of most teacher training programs. However, greater linkage with peer assisted learning activities in the clinical setting would ensure additional practice, with ongoing reinforcement of knowledge and skills. Within the healthcare workforce, teaching is a core professional skill required by health professionals of all levels, from graduates to experienced clinicians, and academics. However, the vast majority of published teacher training program examples are confined to medicine, and were voluntary. Generally, health professionals are not only expected to teach their peers and juniors within their own disciplines, but also across a range of health disciplines, and importantly, their patients. This suggests that students, faculty and institutions may benefits from training programs being designed as relevant across health professions, with the potential for interprofessional implementation; and embedded within curricula. Consistent reporting of teacher training programs, and objective methods of evaluation would enable more in-depth investigation.

## Abbreviations

OSCE: Objective Structured Clinical Examination
PAL: Peer Assisted Learning
